# Glucagon Like Peptide-1 (GLP-1) Modulates OVA-Induced Airway Inflammation and Mucus Secretion Involving a Protein Kinase A (PKA)-Dependent Nuclear Factor-κB (NF-κB) Signaling Pathway in Mice

**DOI:** 10.3390/ijms160920195

**Published:** 2015-08-26

**Authors:** Tao Zhu, Xiao-ling Wu, Wei Zhang, Min Xiao

**Affiliations:** 1 Department of Respiratory Medicine, ZhuJiang Hospital, Southern Medical University, Guangzhou 510280, China; E-Mail: zhutao063020@163.com; 2 Department of Respiratory Medicine, and Division of Pulmonary Diseases, State Key Laboratory of Biotherapy of China, West China Hospital of Sichuan University, Chengdu 610041, China; E-Mail: wuxiaoling2010@126.com; 3 Department of Respiratory Medicine, First Affiliated Hospital of Chengdu Medical College, Chengdu 610500, China; E-Mail: zhangweicdmc@126.com

**Keywords:** asthma, glucagon like peptide-1 (GLP-1), liraglutide, E-selectin, MUC5AC, protein kinase A (PKA), nuclear factor-κB (NF-κB)

## Abstract

Asthma is a common chronic pulmonary inflammatory disease, featured with mucus hyper-secretion in the airway. Recent studies found that glucagon like peptide-1 (GLP-1) analogs, including liraglutide and exenatide, possessed a potent anti-inflammatory property through a protein kinase A (PKA)-dependent signaling pathway. Therefore, the aim of current study was to investigate the value of GLP-1 analog therapy liraglutide in airway inflammation and mucus secretion in a murine model of ovalbumin (OVA)-induced asthma, and its underlying molecular mechanism. In our study, BALB/c mice were sensitized and challenged by OVA to induce chronic asthma. Pathological alterations, the number of cells and the content of inflammatory mediators in bronchoalveolar lavage fluid (BALF), and mucus secretion were observed and measured. In addition, the mRNA and protein expression of E-selectin and MUC5AC were analyzed by qPCR and Western blotting. Then, the phosphorylation of PKA and nuclear factor-κB (NF-κB) p65 were also measured by Western blotting. Further, NF-κB p65 DNA binding activity was detected by ELISA. OVA-induced airway inflammation, airway mucus hyper-secretion, the up-regulation of E-selectin and MUC5AC were remarkably inhibited by GLP-1 in mice (all *p* < 0.01). Then, we also found that OVA-reduced phosphorylation of PKA, and OVA-enhanced NF-κB p65 activation and NF-κB p65 DNA binding activity were markedly improved by GLP-1 (all *p* < 0.01). Furthermore, our data also figured out that these effects of GLP-1 were largely abrogated by the PKA inhibitor H-89 (all *p* < 0.01). Taken together, our results suggest that OVA-induced asthma were potently ameliorated by GLP-1 possibly through a PKA-dependent inactivation of NF-κB in mice, indicating that GLP-1 analogs may be considered an effective and safe drug for the potential treatment of asthma in the future.

## 1. Introduction

Asthma is a very common chronic pulmonary disorder. According to Global Initiative for Asthma (GINA) guidelines, the prevalence of asthma is estimated to be 1% to 18%, affecting more than 200 million people worldwide [1,2]. Asthma is characterized by chronic, persistent airway inflammation and airway remodeling, leading to incompletely-reversible airway obstruction, particularly at late stages [3,4]. Meanwhile, some studies found that extensive and oversecretion of mucus and mucus plug accumulation inside of airway lumen were commonly found in patients with refractory asthma and fatal asthma, resulting in the poor response to bronchodilators in treatment [3,4,5,6]. It is well-known that goblet cells in airway epithelium play a critical role on mucus secretion in asthma [3,4,6,7]. Goblet cell mucus production is in response to many stimulators, such as histamine, epidermal growth factor (EGF), leukotrienes (LTs), IL-4, IL-5, and IL-13, which are synthesized and released by inflammatory cells, including macrophages, eosinophils, mast cells, and Th2 cells [3,4,8].

GLP-1, a peptide hormone mainly synthetized and secreted by L-cells in guts, is essential for the regulation of glycometabolism. GLP-1 analogs, such as liraglutide and exenatide, have been widely used as a safe and effective drug in type-2 diabetes mellitus (T2DM) [9]. Recently, several studies showed that the expression of GLP-1 receptor (GLP-1R) was not only found in pancreas, but also detected in lung and other organs [10,11,12,13,14,15,16,17]. Viby *et al*. [18] confirmed that GLP-1R was highly expressed in lung tissue, both in mice and humans. Viby *et al.* [18] also determined that GLP-1R agonists could improve pulmonary function and survival rates of obstructive lung disease in mice. Nevertheless, the underlying mechanism was still unclear. Otherwise, some data confirmed that GLP-1 possess a potent anti-inflammatory property through a PKA-dependent signaling pathway [19,20,21,22,23,24]. Arakawa *et al.* [24] showed that LPS-induced macrophage activation and TNF-α expression was significantly reduced by GLP-1 analog exendin-4 through PKA/NF-κB signaling pathway. Therefore, the aim of our current study was to explore the property of GLP-1 analog liraglutide in airway inflammation and mucus secretion in a murine model of OVA-induced asthma, and its underlying molecular mechanism.

## 2. Results

### 2.1. GLP-1 Improves Pulmonary Pathological Alterations in OVA-Induced Chronic Asthma

**Figure 1:**
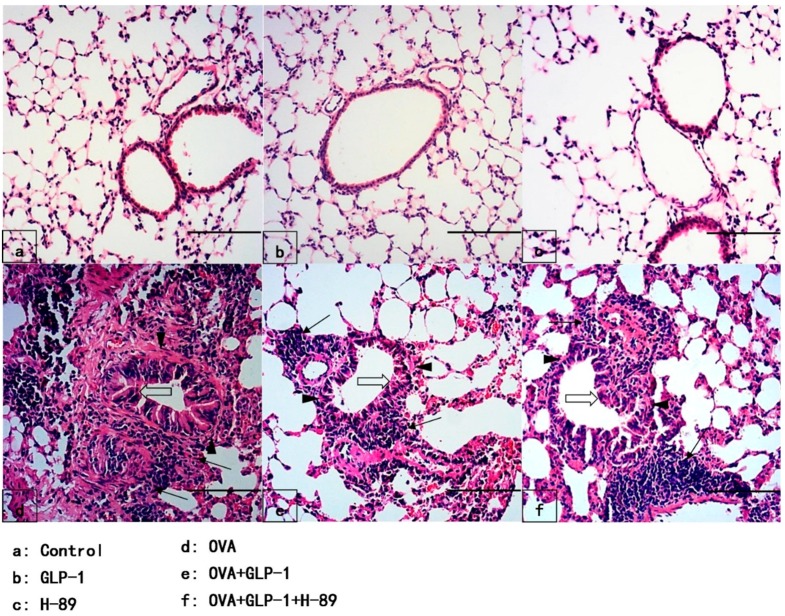
GLP-1 improves pulmonary pathological alterations in OVA-induced chronic asthma. After 81 days of OVA sensitization and challenge, mice were sacrificed and their right lower lungs were fixed. Then, tissue sections were stained with hematoxylin and eosin (H&E). The Figure demonstrates a representative view (×200) from each group. (Inflammatory cells (arrows); Goblet cells (open arrows); Airway basement membrane (triangles)) Scale bar = 100 μm.

To observe the pathological alterations of the lungs, HE staining was performed in our study. After 81 days of OVA sensitization and challenge, the severe and classical pathological alterations were found, which included massive inflammatory cell infiltration, particularly at peribronchial and perivascular areas, significant goblet cell hyperplasia, smooth muscle hyperplasia and hypertrophy, collagen deposition and thicken of the airway basement membrane (Figure 1). However, as shown in Figure 1, the pathological alterations were less severe in the OVA + GLP-1 group. And Figure 1 also demonstrated that the severity of pathological alterations in the OVA + GLP-1 + H-89 group was between the OVA group and the OVA + GLP-1 group, and no pathological change was found in the control group, GLP-1 group and H-89 group (Figure 1).

### 2.2. GLP-1 Reduces Inflammatory Cells Counts in BALF in OVA-Induced Chronic Asthma

**Figure 2:**
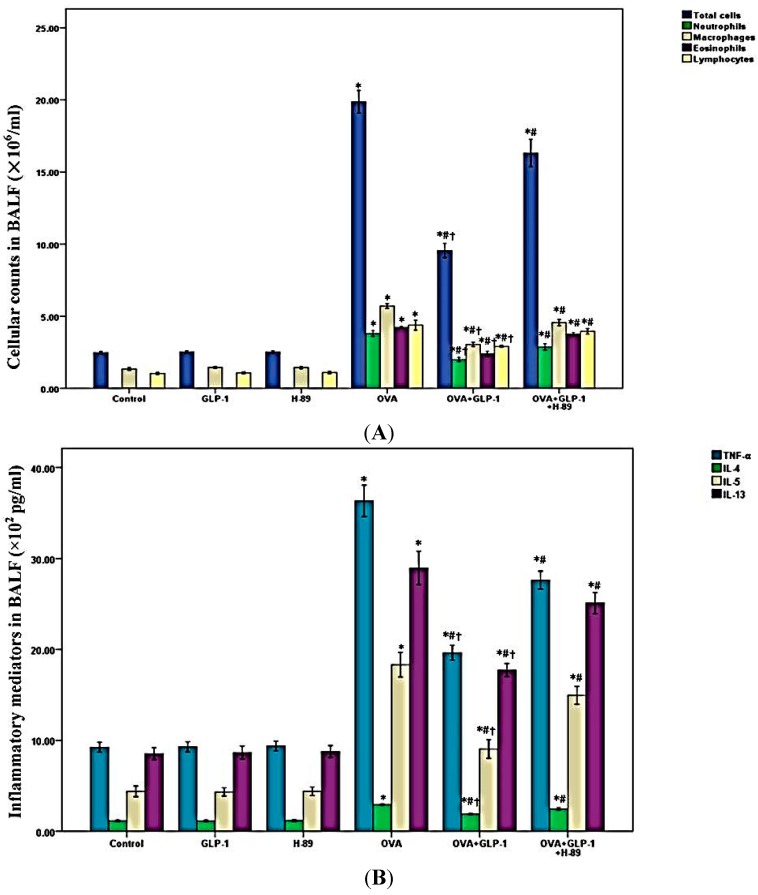
GLP-1 reduces cell counts and inflammatory mediators in BALF in OVA-induced chronic asthma. (**A**) Cells in BALF were collected and cytospin preparations were made. Total cells, neutrophils, macrophages, eosinophils, and lymphocytes in BALF were evaluated; (**B**) TNF-α, IL-4, IL-5, and IL-13 in BALF were detected by ELISA. Each bar represents the mean ± SD of 10 mice. * *p* < 0.05 compared with control. ^#^ *p* < 0.05 compared with OVA. ^†^ *p* < 0.05 compared with OVA + GLP-1 + H-89.

In our study, cell counts in BALF was performed to evaluate the severity of airway inflammation in mice. As shown in Figure 2A, OVA-induced the increment of total cells, neutrophils, macrophages, eosinophils, and lymphocytes in BALF was significantly suppressed by GLP-1. Meanwhile, Figure 2A also demonstrated that this effect of GLP-1 was markedly blunted by H-89.

### 2.3. GLP-1 Decreases Inflammatory Mediators in BALF in OVA-Induced Chronic Asthma

A wealth of inflammatory mediators, particularly the Th2-associated cytokines IL-4, IL-5, and IL-13, and the pro-inflammatory mediator TNF-α, contributed substantially to airway inflammation in asthma [3,25]. As shown in Figure 2B, OVA-induced the over-expression of TNF-α, IL-4, IL-5, and IL-13 in BALF was noticeably decreased by GLP-1. Additionally, we also found that this effect of GLP-1 was largely abrogated by H-89.

### 2.4. GLP-1 Down-Regulates E-Selectin in the Lung Tissues in OVA-Induced Chronic Asthma

**Figure 3:**
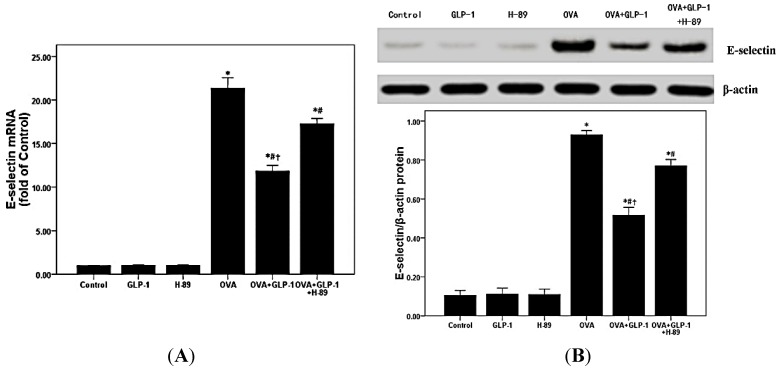
GLP-1 down-regulates E-selectin in the lung tissues in OVA-induced chronic asthma. (**A**) qPCR was used to measure E-selectin mRNA expression in lung; (**B**) Western blotting was performed to measure E-selectin protein expression in lung. Each bar represents the mean ± SD of 10 mice. * *p* < 0.05 compared with control. ^#^ *p* < 0.05 compared with OVA. ^†^ *p* < 0.05 compared with OVA + GLP-1 + H-89.

It is well-known that E-selectin is essential for the recruitment of inflammatory cells, including eosinophils and lymphocytes, in inflamed tissues [26,27]. To explore the mRNA and protein expression of E-selectin, qPCR and Western blotting were performed in our study. According to our data, OVA-induced the up-regulation of E-selectin was notably inhibited by GLP-1 (Figure 3). We also found that this effect of GLP-1 was remarkably blocked by H-89.

### 2.5. GLP-1 Inhibits Airway Mucus Secretion in OVA-Induced Chronic Asthma

**Figure 4:**
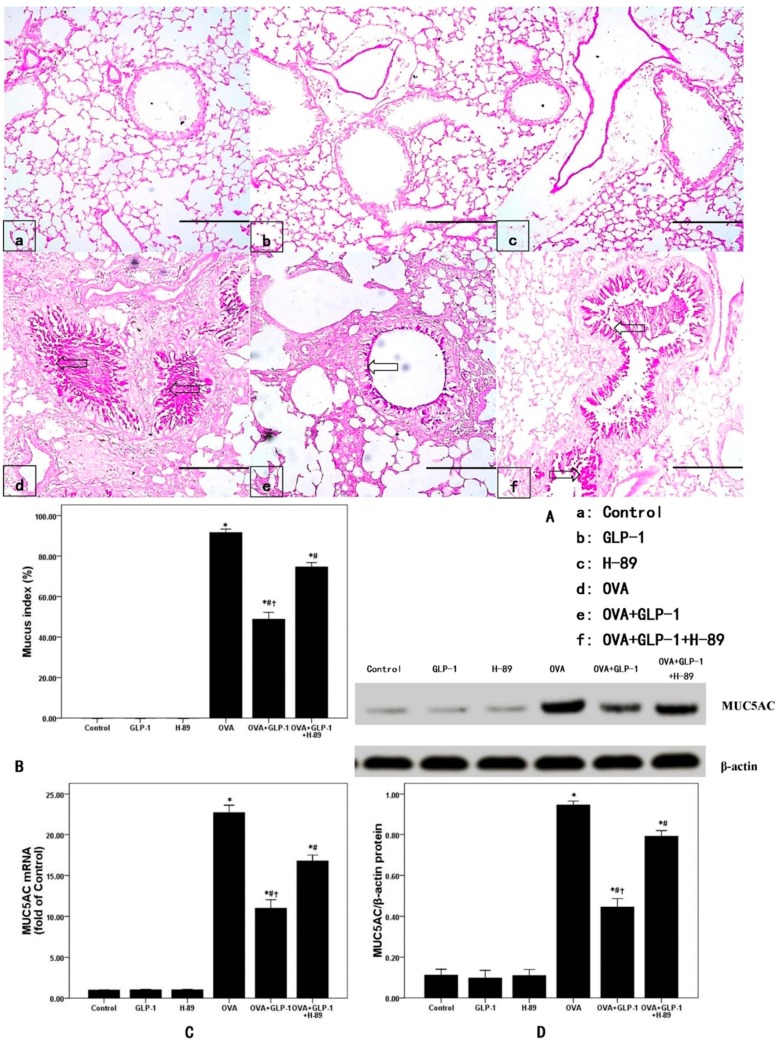
GLP-1 inhibits airway mucus secretion in OVA-induced chronic asthma. (**A**) After 81 days of OVA sensitization and challenge, mice were sacrificed and their right lower lungs were fixed. Then, tissue sections were stained with periodic acid-Schiff (PAS) staining. The Figure demonstrates a representative view (×200) from each group; (Mucus (open arrows)); Scale bar = 100μm; (**B**) the mucus index was calculated as the percentage of the mucus-positive area of the whole bronchial epithelium; (**C**) qPCR was used to measure MUC5AC mRNA expression in lung; (**D**) Western blotting was performed to measure MUC5AC protein expression in lung. Each bar represents the mean ± SD of 10 mice. * *p* < 0.05 compared with Control. ^#^ *p* < 0.05 compared with OVA. ^†^ *p* < 0.05 compared with OVA + GLP-1 + H-89. Scale bar = 100 μm.

Several studies figured out that the majority of airways of fatal asthmatic individuals were blocked by the gelatinous substance composed of mucus and inflammatory exudates [4,6,7]. In our study, mucus secretion and goblet cell hyperplasia were observed and measured by PAS staining and mucus index. As shown in Figure 4A,B, OVA-induced mucus secretion and goblet cell hyperplasia were significantly attenuated by GLP-1. The mRNA and protein expression of MUC5AC, the major component of mucus in the airway, was analyzed by qPCR and Western blotting. Our data showed that OVA-induced the up-regulation of MUC5AC was markedly inhibited by GLP-1 (Figure 4C,D). Then, our data also demonstrated that these effects of GLP-1 were noticeably abrogated by H-89.

### 2.6. GLP-1 Blocks OVA-Induced NF-κB Activation and DNA Binding Activity in a PKA Dependent Manner in Lung

**Figure 5:**
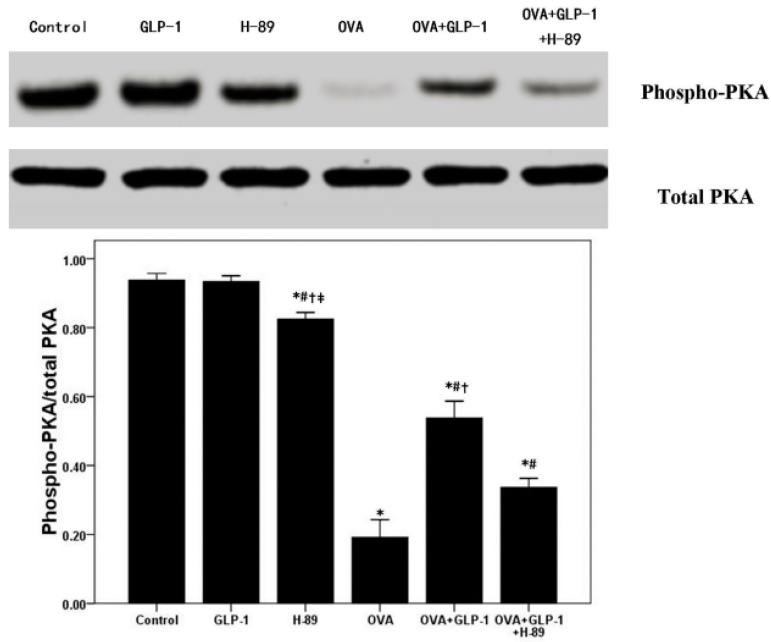
GLP-1 promotes the phosphorylation of PKA in lung in OVA-induced chronic asthma. Western blotting was performed to evaluate the phosphorylation of PKA in the lung tissues. Each bar represents the mean ± SD of 10 mice. * *p* < 0.05 compared with Control. ^#^ *p* <0.05 compared with OVA. ^†^ *p* < 0.05 compared with OVA + GLP-1 + H-89. ^‡^ *p* < 0.05 compared with OVA + GLP-1.

**Figure 6:**
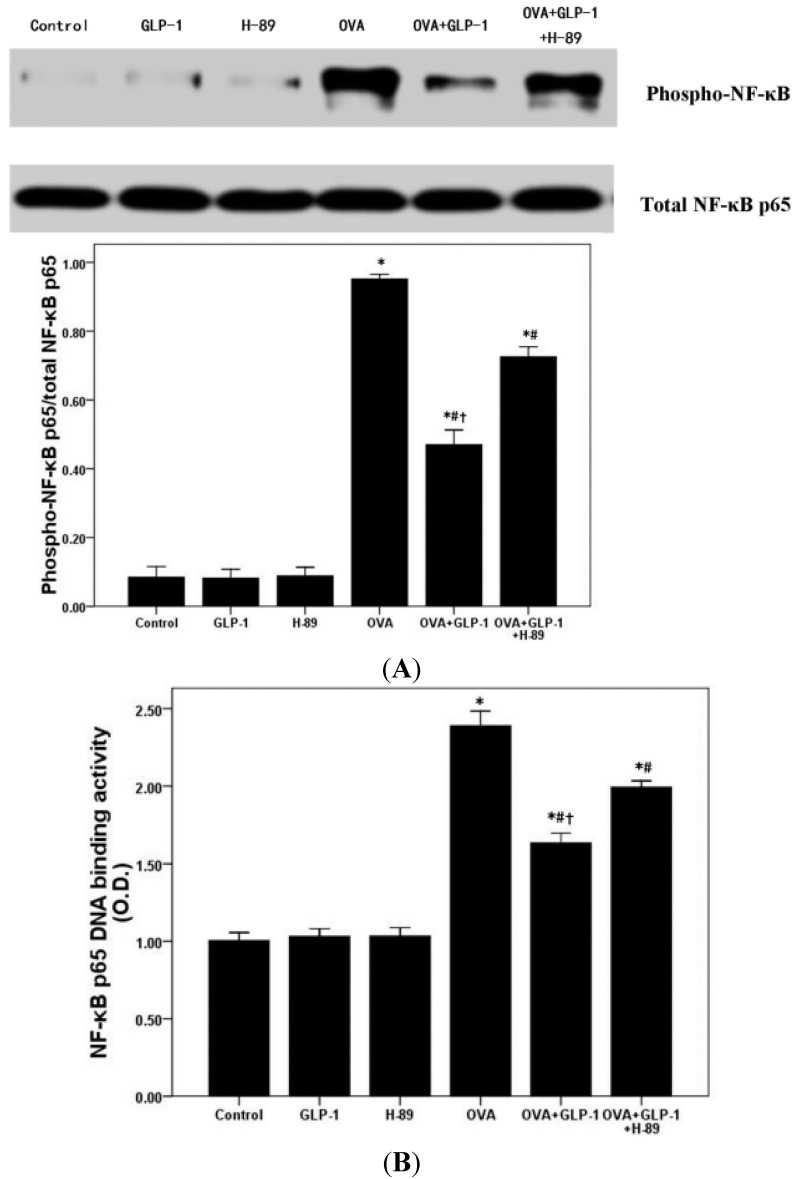
GLP-1 blocks OVA-induced NF-κB activation and DNA binding activity in lung. (**A**) Western blotting was used to analyze the phosphorylation of NF-κB p65 in lung; (**B**) DNA binding activity of NF-κB p65 was measured by a TransAM™ p65 transcription factor ELISA kit. Each bar represents the mean ± SD of 10 mice. * *p* < 0.05 compared with Control. ^#^ *p* < 0.05 compared with OVA. ^†^ *p* < 0.05 compared with OVA + GLP-1 + H-89.

Recently, several studies confirmed that GLP-1 exerted multiple bio-activities through a PKA-dependent signaling pathway, which was also essential for the regulation of NF-κB activation in different inflammatory conditions [19,20,21,24,28]. Firstly, Figure 5 showed that the phosphorylation of PKA was significantly inhibited by OVA sensitization and challenge. GLP-1 markedly compromised OVA-reduced phosphorylation of PKA in the lung. Meanwhile, Figure 5 also demonstrated that this effect of GLP-1 on the phosphorylation of PKA was remarkably blocked by H-89. Furthermore, compared with the control group, the phosphorylation of PKA in H-89 group was slightly reduced. Subsequently, the activation of NF-κB p65 and DNA binding activity of NF-κB p65 were analyzed in our study. Figure 6 showed that OVA-enhanced the activation of NF-κB p65 and NF-κB p65 DNA binding activity were notably abrogated by GLP-1 (Figure 6). Meanwhile, these effects of GLP-1 on the activation of NF-κB p65 and NF-κB p65 DNA binding were also markedly blocked by H-89.

## 3. Discussion

In this study, our results showed that OVA sensitization and challenge induced pulmonary pathological alterations, airway inflammation, the over-expression of E-selectin, and mucus hyper-secretion were markedly ameliorated by GLP-1 in mice. Furthermore, our data also demonstrated that this anti-inflammatory and protective property of GLP-1 was very likely through modulation of a PKA-dependent NF-κB signaling pathway.

Asthma, a common chronic airway disease and featured with reversible airflow obstruction, was caused by the airway allergic reaction with the characteristics of a Th2-predominant airway inflammation [3,8,29]. Many studies have confirmed that airway inflammation laid at the central of the pathogenesis of asthma [4,8]. Then, anti-inflammatory therapy was considered as the fundamental treatment of asthma.

GLP-1, an incretin hormone, was mainly synthesized and secreted by intestinal L-cells [9,30]. It has been confirmed that GLP-1 was essential for the regulation of the level of circulating insulin to stabilize the blood glucose [9,30]. Studies also found that dipeptidyl peptidase-4 (DPP-4) played a key role on GLP-1 metabolism [9,30]. At present, GLP-1 analogs and DPP-4 inhibitors have been widely used in type-2 diabetes mellitus (T2DM) treatment [9,30]. Meanwhile, several studies has identified the expression of GLP-1R in the lung, including airway epithelial cells, type-II alveolar cells and vascular smooth muscle of the pulmonary artery, both in rodents and humans [10,11,12,13,14,15,16,17]. Then, Viby *et al.* [18] showed the protective role of GLP-1R agonists in a mouse model of obstructive lung disease. Nevertheless, the underlying mechanism was unclear. In addition, Wagner *et al.* [31] showed that exendin-4 could stimulate airway mucus secretion in the isolated rat trachea. Nevertheless, the value of GLP-1 analogs in airway mucus secretion needs further investigation. Otherwise, according to the recent studies, in addition to its blood glucose regulation value, GLP-1 analogs and DPP-4 inhibitors also showed potential anti-inflammatory and multiple organs protective properties in different diseases [32,33,34]. Wang *et al.* [35] showed that exendin-4 could improve dyslipidaemia and attenuate atherosclerotic lesion severity and area by reducing macrophage recruitment and activation in female APOE*3-Leiden.CETP mice. Svegliati-Baroni *et al.* [36] determined that exenatide could ameliorate nonalcoholic fatty liver disease (NAFLD)/nonalcoholic steatohepatitis (NASH) through a PKA signaling pathway in rat. After 81 days of OVA stimulation, the severe and classical pathological alterations in lungs were observed. These alterations included massive inflammatory cell infiltration, particularly at peribronchial and perivascular area, goblet cell hyperplasia, smooth muscle hyperplasia and hypertrophy, collagen deposition, and thickening of the airway basement membrane (Figure 1). Meanwhile, our results also showed that these OVA-induced pathological alterations in lungs were less severe in mice with GLP-1 administration. A number of studies proved that macrophages, neutrophils, eosinophils, and lymphocytes were the mainly immunological cells in airway inflammation in asthma [4,8,26]. Our data showed that the number of total cells, macrophages, neutrophils, eosinophils, and lymphocytes in BALF were largely increased after OVA stimulation. We also found that OVA-induced increments of inflammatory cells in BALF were significantly reduced by GLP-1 administration. Furthermore, several reports showed that Th2 cytokines, particularly IL-4 and IL-13, laid at the center of the recruitment of eosinophils and promoting goblet cell metaplasia and hyperplasia, leading to airway mucus hyper-secretion in asthma [25,37,38]. In the current study, our data revealed that OVA induced the raised content of IL-4, IL-5, and IL-13 in BALF was significantly compromised by GLP-1. Therefore, these data suggested that OVA-induced airway inflammation could be remarkably suppressed by GLP-1 in mice.

Many studies showed that goblet cell metaplasia and hyperplasia induced extensive mucus plugs in small airways were commonly observed in autopsy specimens from patients with refractory asthma and fatal asthma, leading to the poor response to bronchodilators and increased airway resistance in the clinic [3,4,5,6,7,39]. Furthermore, the data from morphometric analysis of lung sections showed that goblet cells in small airways in patients with severe asthma increased more than 20-fold compared to healthy individuals [3,4,5,6,7,39]. In the current study, our data determined that the OVA-increased mucus index in the airway was markedly inhibited by GLP-1 (Figure 4A,B). Then, the mRNA and protein expression of MUC5AC, the major component of mucus in the airway, was also analyzed in our study. We found that OVA induced the up-regulation of MUC5AC was also significantly suppressed by GLP-1. Therefore, these findings, in consistent with the previous studies, indicated that GLP-1R should exist on airway epithelial cells [14,15,31]. Our data also suggested that GLP-1 could effectively inhibit mucus hyper-secretion in OVA-induced chronic asthma in mice.

A number of studies confirmed that impaired activation of the cAMP/PKA signal pathway plays a critical role on the pathogenesis of asthma both in pre-clinical and clinical studies [40,41,42,43]. There is increasing evidence that that promoting cAMP/PKA signal pathway activation could relax airway smooth muscle (ASM), reduce airway mucus secretion, suppress inflammatory and immune cells activation and proliferation [40,41,42,43]. Furthermore, phosphodiesterase 4 (PDE4) inhibitors, such as roflumilast, which can inhibit the degradation of cAMP to increase the phosphorylation of PKA in cells, has been widely used in the treatment of COPD and asthma in clinic recently [44,45] and it is well-known that the main therapeutic effects of β2 receptor agonists, such as salmeterol and salbutamol, also result from of the activation of the cAMP/PKA signal pathway [46]. Furthermore, several recent studies determined that the bio-active and pharmacological effects of GLP-1 analogs and DPP-4 inhibitors mainly resulted from the activation of a PKA-dependent signaling pathway [20,22,23]. Dai *et al.* [20] found that liraglutide could noticeably suppress macrophages ox-LDL uptake by inactivation of PKA *in vitro*. In the current study, firstly, our data showed that the effects of GLP-1 on OVA-induced airway inflammation and mucus hyper-secretion were noticeably abrogated by the PKA inhibitor H-89. Otherwise, many studies, including our previous data, confirmed that NF-κB activation, playing a hub role in the regulation of inflammation in different conditions, was involved in the bio-active of GLP-1 [47,48,49,50]. The amount of data also confirmed that PKA was essential for the modulation of NF-κB activation [28,51]. Simultaneously, many studies determined that the *de novo* synthesis of TNF-α and E-selectin, the critical inflammatory molecules, were mainly regulated by NF-κB activation in ARDS and asthma [27,52,53]. Our data showed that OVA induced the raised content of TNF-α in BALF and the up-regulation of E-selectin were remarkably suppressed by GLP-1. Subsequently, we also found that OVA-reduced phosphorylation of PKA and OVA-enhanced NF-κB p65 activation and NF-κB p65 DNA binding activity were all noticeably improved by GLP-1. However, these effects of GLP-1 were all markedly blunted by PKA inhibitor H-89. Therefore, our data suggested that GLP-1 could attenuate OVA-induced NF-κB activation and DNA binding activity via a PKA-dependent manner in mice.

At present, liraglutide, a GLP-1 analog, has been widely used for the treatment of diabetes in clinical therapy. Thus, it has more advantages than a newfound or recently synthesized drug, which needs a very long period and heavy cost to test and evaluate for safety in clinical practice. Nevertheless, clinical studies, particularly randomized-controlled trials (RCTs), should be carried out to further investigate the therapeutic effects of Liraglutide and other GLP-1 analogs in asthma.

## 4. Experimental Section

### 4.1. Animals

All experiments were performed with approval by the West China Medical School of Sichuan University committee on animal policy and welfare, and all efforts were made to minimize animal suffering. We used BALB/c male mice aged 6–8 weeks old (18–22 g) at the beginning of the experiments. Mice were exposed to a 12:12 h dark:light cycle, and offered standard water and chow *ad libitum*.

### 4.2. Murine Model of OVA-Induced Chronic Asthma

Sixty male BALB/c mice were randomly divided into six groups: control group, GLP-1 group, H-89 (the PKA inhibitor) group, OVA group, OVA + GLP-1 group, and OVA + GLP-1 + H-89 group, with 10 in each. According to our previous studies, a murine model of chronic asthma was induced by OVA sensitization and challenge [4]. In brief, mice were sensitized intraperitoneally with 10 μg of OVA (grade V, Sigma-Aldrich Chemical, St. Louis, MO, USA) in 100 μg of Al(OH)_3_ on day 0, 7 and 14. From day 15 to 81, the mice were challenged with 5% OVA aerosol for 1.5 h once daily. Meanwhile, from day 15 to 81, liraglutide (2 mg/kg in 200 µL sterile saline, Novo Nordisk A/S, Novo Alle, DK-2880 Bagsvaeed, Denmark) was given by intraperitoneal injection twice daily. And H-89 (10 mg/kg, Sigma-Aldrich, St. Louis, MO, USA) was also given by intraperitoneal injection once daily. The mice in the control group were sensitized, challenged, and treated with PBS instead.

### 4.3. Cells Counts in BALF

After 81 days of interventions, mice were sacrificed. One hour after OVA challenge on day 81, mice were sacrificed after anesthesia by pentobarbitone (50 mg/kg i.p.) [4]. According to our previous studies, BALF was collected by cannulating the upper part of the trachea, by lavage two times with 1 mL and then 0.8 ml of PBS (pH 7.2). The fluid recovery rate was about 85%–90%. Lavaged sample was kept on ice. BALF was centrifuged at 700× g for 5 min at 4 °C. The sediment cells were resuspended in 50 μL PBS and stained with Diff-Quik (International Reagents Corp., Kobe, Japan) for cytospin preparations. Then, total cells, neutrophils, macrophages, eosinophils, and lymphocytes were counted double-blindly with a hemocytometer [4].

### 4.4. TNF-α, IL-4, IL-5 and IL-13 in BALF

According to our previous study, quantification of the protein levels of TNF-α (Catalog Number: MTA00B), IL-4 (Catalog Number: M4000B), IL-5 (Catalog Number: M5000), and IL-13 (Catalog Number: M1300CB) in BALF were measured by ELISA (R&D Systems, Minneapolis, MN, USA) [4]. Briefly, the BALF supernatant was collected after centrifugation (for 4 min at 4000 r/min) and stored at −70 °C before inflammatory mediators assay. The TNF-α, IL-4, IL-5, and IL-13 assays were carried out according to the manufacturer’s specifications. The limits of detection of IL-4, IL-5, IL-13, and TNF-α were 7.8, 15.6, 7.8, and 10.9 pg/mL, respectively.

### 4.5. H&E Staining and Periodic Acid-Schiff (PAS) Staining

According to our previous study, the histological changes were observed [4]. In brief, the right lower lung tissues were fixed in 10% buffered formalin, embedded in paraffin, sectioned, stained with H&E and examined for pathological changes under light microscopy. We stained mucus and mucus-containing goblet cells in the bronchial epithelium with periodic acid-Schiff (PAS) staining. The histological mucus index (the percentage of the mucus-positive area of the whole bronchial epithelium) was observed and calculated to determine the mucus secretion by two blinded investigators [4].

### 4.6. Quantitative PCR

According to our previous studies, the mRNA expression levels of E-selectin and MUC5AC were detected by qPCR [27,48]. And β-actin was used as internal reference. Briefly, the right upper lung tissues were kept in −80 °C, total RNA was isolated from the lung tissues by Trizol reagent (Invitrogen, Carlsbad, CA, USA). PrimerScript^®^ RT reagent kit with gDNA eraser (Takara Bio Inc., Otsu, Japan) was used to the reverse transcription. Then, PCR was performed with a iQ™5 Multicolor Real-Time PCR Detection System (Bio-Rad Laboratories, Inc., Hercules, CA, USA) and a SYBR Green PCR kit (Takara Bio Inc., Otsu, Japan). The primers and Taqman probes were designed using Primer Premier (PREMIER Biosoft International, Canada). The premier sequences were as follows: E-selectin (forward) 5′-CATGACGTATGATGAAGC-3′ and (reverse) 5′-GATTGGAGTTAAGGTAGTTG-3′; MUC5AC (forward) 5′-GATGACTTCCAGACTATCAGTG-3′ and (reverse) 5′-TGGCGTTAGTCAGCAGA-3′ and β-actin, (forward) 5′-GATTACTGCTCTGGCTCCTAGC-3′ and (reverse) 5′-ACTCATCGTACTCCTGCTTGCT-3′. Changes in the expression of target genes were calculated using the 2**^−^**^ΔΔ^*^C^*^t^ method, ΔΔ*C*t = (*C*t_target_ − *C*t_β-actin_)_sample_ − (*C*t_target_ − *C*t_β-actin_)_control_.

### 4.7. Western Blotting

As described before, the protein expression in lung was measured by western blotting [54,55]. Briefly, protein lysates from left upper lung tissues were run on SDS–PAGE gels and transferred to nitrocellulose membranes. Antibodies against E-selectin (1:500, polyclonal antibody, Catalog Number: sc-14011), MUC5AC (1:500, polyclonal antibody, Catalog Number: sc-16903), phospho-NF-κB p65 (1:800, polyclonal antibody, Catalog Number: sc-101749), NF-κB p65 (1:800, polyclonal antibody, Catalog Number: sc-372), and β-actin (1:1000, polyclonal antibody, Catalog Number: sc-130656) were purchased from Santa Cruz Biotechnology, Inc., Santa Cruz, CA, USA. Furthermore, antibody against phospho-PKA (1:1000, polyclonal antibody, Catalog Number: 4781) and PKA (1:1000, polyclonal antibody, Catalog Number: 4782) were obtained from Cell Signaling Technology, Inc., Danvers, MA, USA.

### 4.8. NF-κB p65 DNA Binding Activity Assay

NF-κB p65 DNA-binding activity was analyzed by TransAM™ NF-κB p65 Chemi Transcription Factor Assay kit (Active Motif, Carlsbad, CA, USA) in our study [54,55].

### 4.9. Statistical Analysis

Data were described as mean ± SD. Comparisons between multiple groups were made using one-way ANOVA, followed by Dunnett test, and pair-wise comparisons between two groups were made using a student’s *T*-test with SPSS software, version 17.0 (SPSS, Inc., Chicago, IL, USA). Then, *p* < 0.05 was considered to be statistically significant.

## 5. Conclusions

Taken together, our data indicated that OVA sensitization and challenge induced airway inflammation and mucus hyper-secretion were potently compromised by GLP-1 through a PKA-dependent NF-κB signaling pathway in mice.
